# Data-driven prediction of the equivalent sand-grain roughness

**DOI:** 10.1038/s41598-023-46564-4

**Published:** 2023-11-04

**Authors:** Haoran Ma, Yuhao Li, Xin Yang, Lili Ye

**Affiliations:** 1 Department of Ocean Engineering, Texas A&M University, College Station, TX 77843 USA; 2 Department of Oceanography, Texas A&M University, College Station, TX 77843 USA; 3 School of Naval Architecture, Ocean and Energy Power Engineering, Wuhan University of Technology, Wuhan, 430063 China

**Keywords:** Engineering, Physics

## Abstract

Surface roughness affects the near-wall fluid velocity profile and surface drag, and is commonly quantified by the equivalent sand-grain roughness ${k}_{s}$. It is essential to estimate ${k}_{s}$ for accurate fluid dynamic problem modeling. While numerous roughness correlation formulas have been proposed to predict ${k}_{s}$ in the fully rough regime, most of them are restricted to certain roughness types, with various geometric parameters considered in each case, leading to ongoing disagreements regarding its parameterization and lack of universality. In this study, a Particle Swarm Optimized Backpropagation (PSO-BP) method is proposed to predict ${k}_{s}$ based on the selected surface parameters from previous DNS, LES, and experimental results for flow behavior over various surface roughness. The PSO-BP model’s ability to predict ${k}_{s}$ in the fully rough region is evaluated and compared with both the existing roughness correction formulas as well as the traditional BP model. An optimized polynomial function is also proposed to serve as a ‘white box’ for predicting ${k}_{s}$. It turns out that the PSO-BP method has better performance in the evaluation metrics compared to other methods, yielding a Mean Absolute Error (MAE) of 0.0390, a Mean Squared Error (MSE) of 0.0026 and a Mean Absolute Percentage Error (MAPE) of 28.12%. This novel approach for estimating ${k}_{s}$ has practical applicability and holds promise for improving the precision and efficiency of calculations related to equivalent sand-grain roughness, and thus provides more accurate and effective solutions for CFD and other engineering applications.

## Introduction

Surface roughness significantly impacts flow-related engineering applications, leading to increased skin friction compared to smooth surfaces^1^, and results in a downshift of near-wall velocity profile which can be quantified by:

1 $${U}^{+}=\frac{1}{\kappa }ln{y}^{+}+B-\Delta {U}^{+}$$

where $\kappa \approx 0.4$ is the von Kármán constant, $B\approx 5.0$ is the intercept for the smooth wall case and the roughness function $\Delta {U}^{+}$ is related to roughness Reynolds number ${k}_{s}^{+}$ in the fully rough regime via:

2 $${\Delta U}^{+}=\frac{1}{\kappa }ln{k}_{s}^{+}+B-8.5$$

in which ${k}_{s}^{+}=\frac{{k}_{s}{U}_{\tau }}{\nu }$ and ${k}_{s}$ denotes the equivalent sand-grain roughness. And it is conceptualized as an equivalent roughness height for surfaces that are irregular and non-uniform, yielding the same skin-friction coefficient as the uniform sand-grain of the Nikuradse’s in the fully-rough regime^2^. When simulating the near-wall flow using Reynolds-Averaged Navier–Stokes (RANS) method in Computational Fluid Dynamics (CFD), surface roughness effects can be accounted for by introducing the equivalent sand-grain roughness length scale ${k}_{s}$ into the boundary condition of turbulence models^3^. However, this approach necessitates the a priori knowledge of the ${k}_{s}$ value.

There exist numerous correlations for the equivalent sand-grain roughness ${k}_{s}$. Chan^4^ confirmed strong correlation exists between roughness function and mean roughness height ${k}_{a}$ as well as streamwise effective slope $ES$ over sinusoidal roughness; Forooghi^5^ found that a combination of mean maximum peak-to-trough height ${k}_{t}$, skewness $Sk$ and effective slope $ES$ can lead to satisfactory prediction of ${k}_{s}$ over randomly distributed roughness elements; Flack^6^ proposed a roughness correlation formula based on the root-mean-square roughness height ${k}_{rms}$ and skewness ${(S}_{k})$ from various grit blasting surfaces, and Kuwata^7^ developed a roughness correlation function also based on ${k}_{rms}$ and ${S}_{k}$ over randomly distributed hemispheres, to name a few. Although previous research provided valuable insights into the correlation between surface features and the equivalent sand-grain roughness, various geometrical parameters are considered in each roughness correlation formula. Moreover, these correlation formulas are limited to the particular roughness types they investigated, suggesting a potential lack of universality that might constrain the effectiveness when applied to arbitrary surface roughness. Therefore, it is crucial to establish a universal roughness correlation that leverages essential surface features, minimizing complexity while maintaining applicability to a wide variety of roughness types, and also enable the accurate prediction of ${k}_{s}$.

Despite the difficulty of deriving the relationship in the traditional approach, the machine learning (ML) technique is helpful when dealing with the high-order features problem and mapping the complex relationship between surface parameters to equivalent sand-grain roughness. Jouybari^8^ used Deep Neural Network (DNN) and Gaussian Process Regression (GPR) methods to train the model and predict ${k}_{s}$ with 17 inputs containing both roughness parameters and their products. Even though they achieved relatively low errors compared with existing roughness correlation formulas, these methods have their shortcomings. For example, only 45 different datasets with 17 inputs considered in their study could lead to overfitting and limit DNN’s reliability since it’s typically reliant on large datasets. Also, the performance of GPR heavily depends on the choice of kernel function and its hyperparameters, which often require further experimentation or cross-validation, and selecting an inappropriate kernel can lead to poor model performance. Furthermore, it took approximately 75 h to obtain the optimal DNN network and 35 h for the GPR method, which is time-consuming.

The Backpropagation (BP) neural network has its advantages like non-linear mapping ability and simple structure with high efficiency. And Particle Swarm Optimization (PSO) is a global optimization technique inspired by the social behavior of bird flocks, and it can help to find a better set of weights and biases for the BP neural network by exploring the search space more thoroughly and reducing the chance of being stuck in the local minimum. In this research, a Particle Swarm Optimized Backpropagation (PSO-BP) neural network is proposed to attempt to achieve a more universal correlation between the most important features (to be analyzed in the next section) and equivalent sand-grain roughness over various rough surface types, ranging from hemispheres, truncated cones, numerically generated surfaces or replicated from real engineering applications, in the fully rough regime. It turns out the PSO-BP method outperforms selected roughness correlation formulas and traditional BP algorithm as well as the optimized polynomial function, hence providing an effective method for estimating ${k}_{s}$ under various types of roughness.

## Methods

### Surface parameters analysis

According to Flack^9^ an ideal model for predicting equivalent sand-grain roughness ${k}_{s}$ should be based on readily measurable surface parameters, which could be determined via a simple surface scan, thus avoiding the need for further experiments or DNS simulations. Since it’s not possible to take all surface geometric features into consideration when predicting ${k}_{s}$, it’s necessary to find a minimal yet representative set of surface parameters that can sufficiently convey the drag-producing characteristics over various roughness types^9^.

Chung^10^ suggested that a robust correlation between surface topography and equivalent roughness requires the consideration of at least three categories. The first one encompasses surface elevation, which can be represented by parameters such as $${k}_{a},{k}_{t},{k}_{rms}$$, etc. The second involves the effective slope ($$ES)$$, the frontal solidity $$({\lambda }_{f})$$ or the roughness density parameter ($${\Lambda }_{s})$$. Finally, the third component is characterized by plan solidity $$({\lambda }_{p})$$, solid volume fraction $$(\phi )$$, or the skewness $$(Sk)$$.

When it comes to representing roughness height, the root-mean-square height ${k}_{rms}$ gives a measure of the standard deviation of the height distribution^10^,which means it takes both the amplitude and distribution of surface asperities into account, providing a more comprehensive statistical measure of the surface texture over the former two roughness height, namely, the average roughness height ${k}_{a}$ and the mean maximum peak-to-trough roughness height ${k}_{t}$. As different surface structures can possess the same ${k}_{a}$ value and ${k}_{t}$ is more sensitive to occasional high peaks or deep valleys, both of which can lead to misinterpretation of the surface characteristics.

And the effective slope $ES$ stands for the mean absolute streamwise gradient of the surface roughness and provides a measure of the steepness of roughness features^9^. It’s worth noting that the frontal solidity $({\lambda }_{f})$, and roughness density parameter (${\Lambda }_{s})$ are closely related to the effective slope $ES$, with $ES=2{\lambda }_{f}$ and ${\Lambda }_{s}$ is related to the local angle ${\alpha }_{h}$ whose absolute value is approximately $ES$^10^. The skewness $Sk$ reveals whether the surface is dominated by peaks ($Sk>0$) or pits ($Sk<0$)^9^. In addition, plan solidity $({\lambda }_{p})$, which is defined as the ratio of plan area, and solid volume fraction $(\phi )$, are also have a strong correlation with $Sk$. Besides, Bhaganagar and Chau^11^ found that the kurtosis $Ku$, which accounts for the sharpness of the height distribution of a surface, could be effective for characterizing the roughness function from their DNS results. The mathematical expressions for ${k}_{rms}$, $ES$, ${\lambda }_{f}$, ${\Lambda }_{s}$, ${\lambda }_{p}$, $Sk$ and $Ku$ are provided in the Supplementary Materials.

Other than the above analysis, the correlation heatmap can offer useful perspective into the relationships among different parameters, particularly in identifying the most influential factors on a given outcome. Thus, a correlation heatmap is created based on data provided by Jouybari^8^ where 45 distinct roughness geometries were considered with 12 surface parameters investigated. The color intensity and numeric values within each cell represent the correlation coefficient between variable pairs.

**Figure 1:**
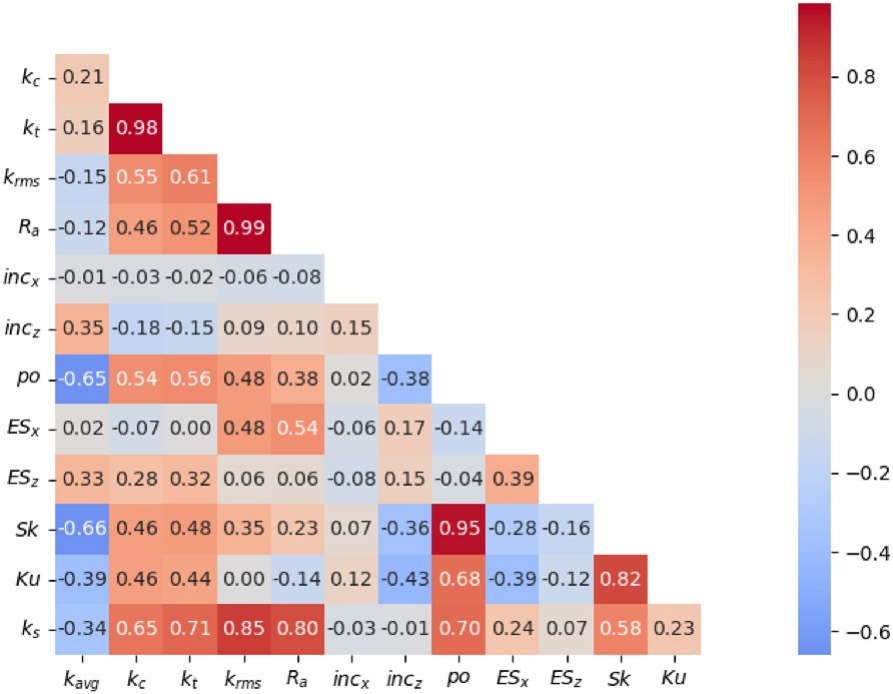
Heatmap of the correlation matrix.

The geometric parameters shown in Fig. 1 are: mean roughness height $${k}_{avg}$$, roughness peak-to-trough height $${k}_{c}$$, mean maximum peak-to-trough height $${k}_{t}$$, root-mean-square roughness height $${k}_{rms}$$, first-order moment of height fluctuations $${R}_{a}$$, inclination angle in $$i$$ directions $${inc}_{i}$$, surface porosity $${P}_{o}$$, effective slope in $$i$$ directions $${ES}_{i}$$, skewness $$Sk$$ and kurtosis $$Ku$$, respectively. Among the roughness height related parameters ($${k}_{avg},{k}_{c},{k}_{t},{k}_{rms},{R}_{a})$$, $${k}_{rms}$$ shows the strongest correlation with $${k}_{s}$$ and has a coefficient of 0.85, indicating that as $${k}_{rms}$$ increases, the equivalent sand-grain roughness likely increases significantly. Also, $$Sk$$ and $${ES}_{x}$$ both exhibit moderate positive correlations with $${k}_{s}$$, coefficients being 0.58 and 0.24 respectively, suggesting that their increase is associated with an increase in $${k}_{s}$$, and the streamwise $$ES$$ is more involved than spanwise. Similarly, $$Ku$$ also shows a positive correlation with $${k}_{s}$$ (coefficient of 0.23). Since the limited data of surface porosity $${P}_{o}$$ and it also exists strong correlation with $$Sk$$ (0.95), we don’t take $${P}_{o}$$ into further consideration.

The correlation heatmap result substantiates the previous analysis, and hence we adopted the root-mean-square roughness height ${k}_{rms}$, streamwise effective slope $ES$, skewness $Sk$ and kurtosis $Ku$ as the representative surface parameters to serve as the input variables for predicting equivalent sand-grain roughness ${k}_{s}$ through the BP and PSO-BP methods.

### Particle swarm algorithm and neural network

Backpropagation (BP) is a prominent machine learning algorithm achieved by repeated forward propagation of input and backward propagation of error procedure with an iterative adjustment of weights and biases, until the output error is reduced to an acceptable level or the preset maximum iteration is reached. The BP neural network (BPNN) is well-suited for approximating nonlinear functions with multivariate input features while still maintaining a simple structure. However, the BPNN does come with certain drawbacks, such as the sensitivity to random initial weights and biases, and it’s not guaranteed to find the global minimum. Instead, it may get stuck in a local minimum.

And the particle swarm optimization (PSO) is a population-based stochastic optimization technique inspired by the social behavior of bird flocking, where birds adjust their navigation based on individual experience and group communication to help the entire flock get the best hunt^12^. In the PSO technique, each particle is a potential solution to the target problem and has a fitness value which is essentially a score that indicates how well the particle meets the objective function of the optimization problem. And it is obtained from the fitness function by the Root Mean Squared Error (RMSE) calculated through each training set in this particular case:

3 $$fitness=\sqrt{\frac{1}{n}\sum_{i=1}^{n}{({y}_{i}-\widehat{{y}_{i}})}^{2}}$$

where ${y}_{i}$ is the actual output value and $\widehat{{y}_{i}}$ is the predicted output value.

During each iteration, the velocity of each particle is updated towards their previous individual optimal position and global optimal position in a multidimensional search space, and then the particle position is updated according to the new velocity. After satisfying the predefined criterion, it jumps out of the iteration thus obtaining the optimal solution. The equations of particle update operations are as follows (Shi^13^):

4 $${V}_{iD}^{j+1}=\omega {V}_{iD}^{j}+{c}_{1}{r}_{1}\left({p}_{iD}^{j}-{x}_{iD}^{j}\right)+{c}_{2}{r}_{2}\left({p}_{gD}^{j}-{x}_{iD}^{j}\right)$$

5 $${x}_{iD}^{j+1}={x}_{iD}^{j}+{v}_{iD}^{j+1}$$

where $i$ stands for the particle and $j$ denotes the current iteration number; $D$ is the particle search dimension; ${x}_{iD}^{j}$ and ${v}_{iD}^{j}$ are the particle position and velocity in the $j$ iteration; non-negative acceleration factor ${c}_{1}$ represents the individual’s cognitive ability and ${c}_{2}$ represents the social guidance of the group; ${r}_{1}$ and ${r}_{2}$ are independently evenly distributed random numbers in the interval (0, 1); $\omega$ is the inertia weight which controls the impact of previous particle velocity on its current one in the solution domain, which is achieved by:

6 $$\omega ={\omega }_{max}-\frac{j}{{j}_{max}}({\omega }_{max}-{\omega }_{min})$$

in which ${j}_{max}$ is the maximum iteration number. And this formula indicates that the inertia weight linearly decreases from ${\omega }_{max}$ to ${\omega }_{min}$ as the PSO technique progresses from the first iteration to the last one. It allows the algorithm to initially explore the search space in a wide range and then gradually focus more on fine-tuning the best-found solutions, hence achieving a balance between exploration and exploitation.

And the incorporation of PSO with the BP neural network offers several advantages that can address certain limitations of a traditional BP neural network. Primarily, PSO is a global optimization method, which can reduce the chance of the BP neural network getting stuck in local minima during the learning process. Also, PSO can facilitate a faster convergence of the learning process, and reduce the computational time required to train the neural network. Furthermore, by finding a more global solution, the PSO-BP method can reduce the risk of overfitting, which is also a common problem in the neural network training process. Lastly, PSO provides a more robust approach with few parameters to adjust and makes the BP neural network less dependent on the initial conditions since it will provide the global optimal values to BP.

**Figure 2:**
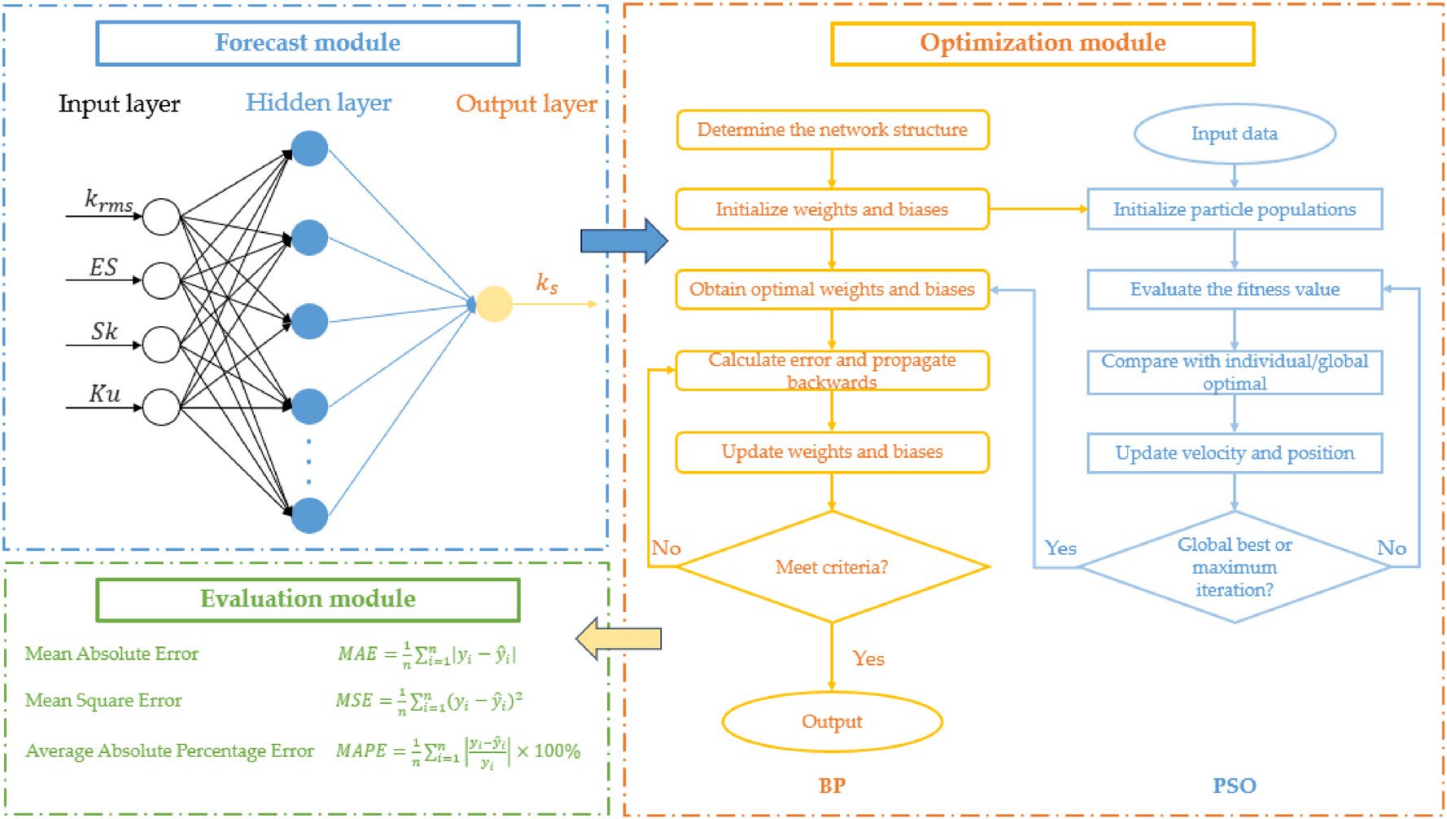
Overall prediction module of the PSO-BP algorithm.

The PSO-BP method’s procedure can be expressed as follows with the overall prediction module also shown in Fig. 2:

1. Define the architecture of the BP neural network from the dataset.
2. Initialize weights and biases in the BP neural network.
3. Initialize the particle velocity, position, individual optimal value and global optimal value in the PSO algorithm.
4. Evaluate the particle fitness value individually according to the fitness function. The individual optimal value is updated if the current value is better. And the same approach is executed for the global optimal value if the individual optimal is better.
5. Calculate the new particle velocity, position as well as perform the mutation operation.
6. Return to step (4) if the current number of iterations is less than the maximum iteration value or the error parameter is larger than the set error value.
7. Utilize the obtained optimal weights and biases from the PSO process to train the BP neural network.
8. Calculate the output error through the loss function, propagate the error backward and adjust the weights and biases that minimize the error.
9. Repeat step (8) until the network’s prediction result meets certain criteria.

According to Feng^14^, the equation $2i+1$ provides an upper bound for the number of hidden neurons needed in a one-hidden-layer BP neural network, where $i$ denotes the number of inputs. In this research, we utilized a 4-9-1 three-layer neural network structure for both the BP and PSO-BP methods. We examined a total of 18 combinations of PSO hyper-parameters as shown in Table 1 and the optimal set is highlighted in bold.

**Table 1:** Range of hyper-parameters tested.

Acceleration factors ${{c}_{1}, c}_{2}$	1.49445, **2.0**
Population size	**30**, 40, 50
Evolutionary generation	30, 40, **50**

Optimal values are in bold.

Thus, the PSO hyperparameters are set as follows: acceleration factors ${c}_{1}$ = ${c}_{2}$  = 2.0, population size is 30, evolutionary generation equals 50. The intervals of particle position and velocity are [− 5, 5] and [− 1, 1], ${\omega }_{max}$ and ${\omega }_{min}$ are set to be 0.9 and 0.4, respectively.

### $K$-fold cross-validation

$K$-fold cross-validation is a resampling method used to evaluate machine learning models, especially useful for the current case where data samples are limited. It not only provides a more robust evaluation compared to a single train but also helps reduce overfitting. This procedure begins with the shuffling of original dataset, followed by its division into $k$ subsets (or folds) of approximately equal size. Through each iteration, $k-1$ folds are used for training while the remaining one is allocated for testing. This process is repeated $k$ times, ensuring each fold is used for test exactly once. After completing $k$ iterations, $k$ distinct models and their corresponding evaluation metrics are constructed, and their averaged results can provide a comprehensive assessment of the model’s capabilities. In this study, we employed the fivefold cross-validation, and the optimal as well as averaged results are presented subsequently.

### Model evaluation indicators

Evaluating the performance of the PSO-BP method is a crucial step, providing an understanding of the reliability of our predictions and aiding in the comparison of different models. In this study, we employed four key evaluation metrics: coefficient of determination (${R}^{2}$), Mean Absolute Error (MAE), Mean Squared Error (MSE), and Mean Absolute Percentage Error (MAPE) to evaluate the existing roughness correlation formulas, the trained results from PSO-BP and traditional BP methods, as well as the optimized polynomial functions. And the expressions for these metrics are as follows:

7 $${R}^{2}=1-\frac{\sum_{i=1}^{n}({{y}_{i}-{\widehat{y}}_{i})}^{2}}{\sum_{i=1}^{n}({{y}_{i}-\overline{{y }_{i}})}^{2}}$$

8 $$MAE=\frac{1}{n}\sum_{i=1}^{n}\left|{y}_{i}-{\widehat{y}}_{i}\right|$$

9 $$MSE=\frac{1}{n}{\sum_{i=1}^{n}({y}_{i}-{\widehat{y}}_{i})}^{2}$$

10 $$MAPE=\frac{1}{n}\sum_{i=1}^{n}\left|\frac{{y}_{i}-{\widehat{y}}_{i}}{{y}_{i}}\right|\times 100\%$$

among the aforementioned formulas, $n$ is the total number of data points, ${y}_{i}$ represents the actual value, ${\widehat{y}}_{i}$ denotes the corresponding prediction value and $\overline{{y }_{i}}$ is the mean of actual values.

## Results

### Data source and pre-processing

The database utilized for training the BP and PSO-BP neural networks as well as the roughness correlation formulas, consist of direct numerical simulation (DNS) results from Forooghi^5^, Kuwata^7^, Jouybari^8^, Jelly^15^, large eddy simulation (LES) results from Marchis^16^, along with experimental results from Flack^6,17,18^ and Womack^19^, which is totally comprised of 86 distinct datasets and all of them reside within the fully rough regime. The roughness types cover a broad scale, including both regular and irregular, 2D and 3D roughness, ranging from ellipsoidal elements, sand-grain, turbine blade roughness, to grit blasting, truncated cone roughness, and so on. Here, ${k}_{rms}$ and ${k}_{s}$ values from DNS and LES results are all normalized by the channel half-height $\delta$, while those values from Womack^19^ are normalized by the boundary layer thickness. The ranges of the investigated parameters are as follows: $0.01<{k}_{rms}<0.12,$ $0.07<ES<1.14$, $-1.44<Sk<3.96$, $1.43<Ku\le 26.8$. A subset of the sample data is presented in Table 2, while the complete dataset can be found in the Supplementary Materials.

**Table 2:** Partial sample data which are all in the fully rough regime.

${k}_{rms}$	$ES$	$Sk$	$Ku$	${k}_{s}$
0.0219	0.5949	− 0.1671	1.6007	0.0644
0.0326	0.9158	0.1093	1.4355	0.1244
0.0218	0.5876	− 0.0874	1.5898	0.0589
…	…	…	…	…
0.0280	0.3400	0.0100	3.0000	0.1439
0.0280	0.3400	0.0100	2.9800	0.1688
0.0310	0.3500	− 0.0200	2.9400	0.2062

Next, we conducted a feature importance analysis using the Random Forest model which gives an indication of how useful or valuable each feature is toward the model’s predictive performance. This analysis not only supplements the insights gained from the previous heatmap, but also deepens our understanding of the complex interplay between those features especially for our dataset. It allows us to identify the most influential features among those four input variables, particularly when the dataset contains a variety of roughness types. Above all, this analysis guides the selection of appropriate roughness correlation formulas following the feature importance.

**Figure 3:**
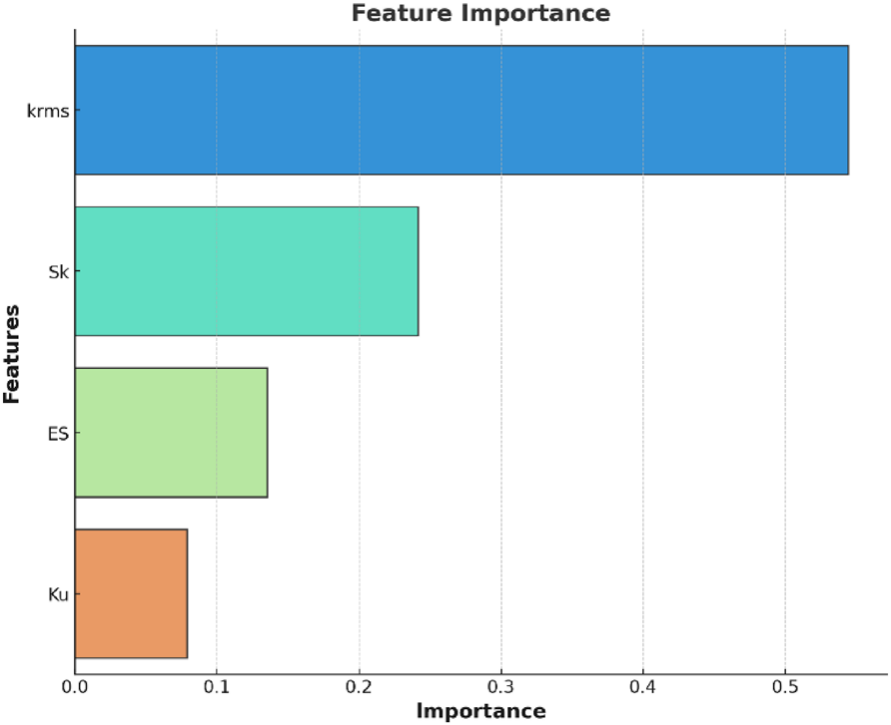
Feature importance plot measured by Random Forest.

As illustrated in Fig. 3, ${k}_{rms}$ emerges as the most influential feature, contributing significantly to the prediction of ${k}_{s}$. It is followed by $Sk$, $ES$ and $Ku$, in descending order of importance. Thus, roughness correlation formulas prioritizing $ES$ are set aside (e.g., Bons^20^, Abdelaziz^21^, Marchis^16^), and we opt for roughness correlation formulas from Flack^6^ and Kuwata^7^, in which the prediction of ${k}_{s}$ rely heavily on ${k}_{rms}$ and $Sk$. Besides, the roughness correlation equation from Musker^22^, which accounts for ${k}_{rms}$, $Sk$, $ES$ and $Ku$ simultaneously is considered. The selection of these correlation formulas aims to enhance the prediction accuracy for the given dataset, allowing for a more equitable comparison with neural networks in subsequent sections.

### Prediction with established correction formulas

Firstly, we employed the roughness correlation formula proposed by Flack^6^ with sample data, given as

11 $${k}_{s}=2.91{k}_{rms}{(2+Sk)}^{-0.284}$$

and compare the predicted values with the actual ones. The obtained evaluation metrics to quantify the performance of this correlation formula are as follows: MAE is 0.1034, MSE is 0.0183, and MAPE is 51.66%.

Secondly, we utilized the formula developed by Kuwata^7^:

12 $${k}_{s}=4.0{k}_{rms}{(1+0.17Sk)}^{4}$$

and in this case, the calculated MAE is 0.0729, MSE is 0.0141, and MAPE is 56.91%.

Lastly, Musker^22^ ’s equation has the form:

13 $${k}_{s}={k}_{rms}(1+0.5ES)(1+0.2Sk \cdot Ku)$$

where the calculated MAE is 0.1316, MSE is 0.0255, and MAPE is 76.22%.

Based on the evaluation metrics, Kuwata’s roughness correlation formula demonstrates superior performance in terms of accuracy, evidenced by the lowest MAE and MSE values under the given dataset, though it shows relatively higher MAPE than Flack’s result. One possible reason is that the rough surfaces generated by randomly distributed hemispheres in Kuwata’s case make it more general and enhance its ability to capture varied surface characteristics. On the other hand, Flack’s correlation formula is based on grit blasting and Musker’s equation considers ship-hull roughness, such specificity might limit their adaptability compared to Kuwata’s approach.

### Prediction with BP neural network

**Figure 4:**
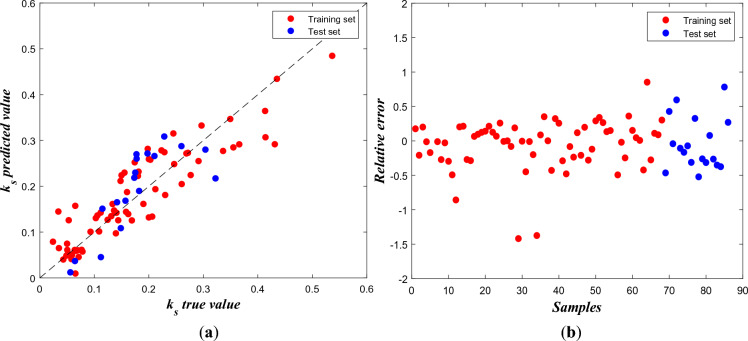
(**a**) Scatter plot of true ${\mathrm{k}}_{\mathrm{s}}$ and predicted ${\mathrm{k}}_{\mathrm{s}}$, (**b**) Scatter plot of relative error for each sample.

The optimal prediction results using BP neural network are evaluated through the scatter plots of true ${k}_{s}$ vs. predicted ${k}_{s}$ values and scatter plot of relative error as well as the evaluation metrics (based on the test set):

and the calculated MAE is 0.0499, MSE is 0.0033 and MAPE is 31.84%. It’s worth noting that in the ${k}_{s}$ true vs. ${k}_{s}$ predict values plot (left), the training and test sets overlap because the prediction results for both sets are close in those regions. On the other hand, the relative error plot (right)’s horizontal axis only represents sample size, and the test set is positioned after the training set for better visualization. As shown by Fig. 4a, most of the points follow the general trend of the regression line, while some deviations exist. Meanwhile, the relative error plot in Fig. 4b exhibits an overall symmetric pattern around zero, indicating the BP neural network’s predictions are, on average, fairly accurate. But there are a few extreme values exist that represent outliers.

### Prediction with PSO-BP neural network

**Figure 5:**
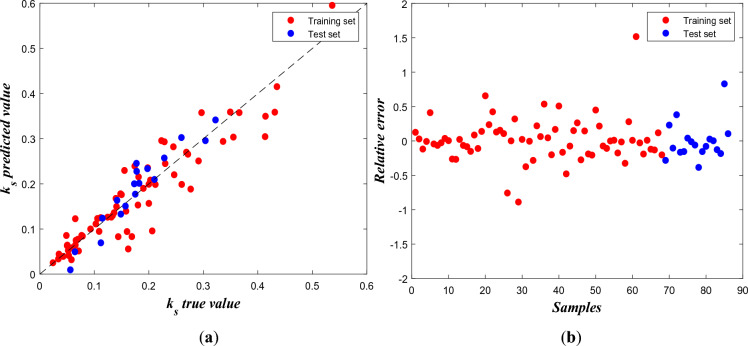
(**a**) Scatter plot of true ${\mathrm{k}}_{\mathrm{s}}$ and predicted ${\mathrm{k}}_{\mathrm{s}}$, (**b**) Scatter plot of relative error for each sample.

The optimal prediction results using PSO-BP neural network are evaluated as follows: and the MAE, MSE and MAPE have been calculated as 0.0255, 0.0010, and 18.46%, respectively. As depicted in the left plot of Fig. 5, data points, in general, lie closer to the regression line than in the BPNN scatter plot. The PSO-BPNN yields more accurate prediction results especially for small ${k}_{s}$ values, while larger ${k}_{s}$ values present some discrepancy from the actual values. One potential explanation for this inconsistency may lie in the limited number of data points for large ${k}_{s}$ values in the dataset. By incorporating additional samples representing larger ${k}_{s}$ values, it might be possible to further increase the model’s accuracy, thereby reducing the observed discrepancy. In addition, the overall relative error values are closer to zero which means the magnitude of errors is reduced, meanwhile, extreme values are alleviated.

**Figure 6:**
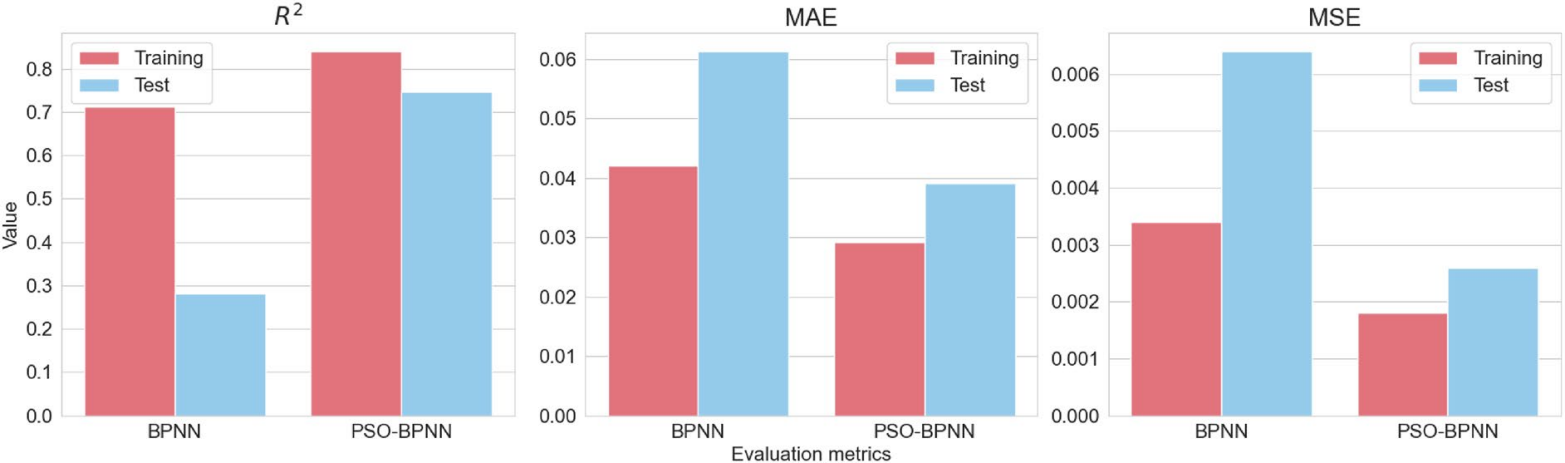
Bar plots of performance evaluation metrics.

The outcomes of the fivefold cross-validation process are presented in Table 3. To provide a more synthesized perspective, the averaged evaluation metrics of BPNN and PSO-BPNN are intuitively represented through bar plots in Fig. 6.

**Table 3:** Statistics of cross-validation evaluation index.

No	BPNN						PSO-BPNN					
	*R*^2^		MAE		MSE		*R*^2^		MAE		MSE	
	Train	Test	Train	Test	Train	Test	Train	Test	Train	Test	Train	Test
1	0.816	0.319	0.0384	0.0499	0.0024	0.0033	0.853	0.794	0.0316	0.0255	0.0019	0.0010
2	0.619	0.055	0.0505	0.0738	0.0044	0.0095	0.831	0.883	0.0306	0.0280	0.0020	0.0012
3	0.496	0.515	0.0515	0.0701	0.0050	0.0076	0.791	0.753	0.0277	0.0521	0.0021	0.0039
4	0.759	0.700	0.0378	0.0474	0.0025	0.0045	0.903	0.713	0.0220	0.0488	0.0010	0.0043
5	0.870	-0.18	0.0319	0.0654	0.0016	0.0072	0.823	0.593	0.0337	0.0407	0.0021	0.0025
Average	0.712	0.282	0.0420	0.0613	0.0034	0.0064	0.840	0.747	0.0291	0.0390	0.0018	0.0026

and the averaged MAPE for BPNN and PSO-BPNN are 46.07% and 28.12%, respectively. The comparison between BPNN and PSO-BPNN reveals critical insights into the efficacy of incorporating particle swarm optimization into BPNN. An obvious observation is the stark contrast in the averaged *R*^2^ values. BPNN, although performing satisfactorily during the training set, exhibits a drastic deterioration in the test phase with an averaged *R*^2^ value of 0.282. This decrease is notably attributed to the *R*^2^ value falling below 0 in the 5th cross-validation of BPNN’s test set, indicating the BPNN fails to capture the underlying data pattern. This could be due to the existence of outliers or extreme values in the test set, which are unrepresented in the training set, thereby skewing the model’s predictive capability.

Conversely, PSO-BPNN demonstrates commendable robustness and generalizability. This is evident not just in the marginal reduction in averaged *R*^2^ from training to testing, but also in its resilient *R*^2^ value of 0.593 in the 5th cross-validation of the test set. Such consistency is indicative of the model's enhanced capacity to mitigate overfitting, likely attributable to the particle swarm optimization's role in efficiently navigating the solution space for optimal weights and biases. Moreover, PSO-BPNN consistently outperforms BPNN across other evaluation metrics as well, as evidenced by its lower MAE, MSE and MAPE in nearly every iteration, thereby implying a higher degree of accuracy and reliability.

### Optimized polynomial model

While the ‘black box’ PSO-BPNN method exhibits satisfying results in predicting the equivalent sand-grain roughness ${k}_{s}$ in the fully rough regime across variety of roughness types, the utilization of explicit algebraic representations, such as using polynomial functions to fit complex nonlinear models provides an alternate intuitive approach for the prediction of ${k}_{s}$. The appeal of this approach lies in its transparency and interpretability, and this is achieved through the curve-fitting procedure, where a basis function is first proposed for the model, and the unknown coefficients of the function are subsequently optimized under certain predefined constraints. In this study, an optimized polynomial function based on Musker^22^ ’s roughness correlation type has been proposed as a ‘white box’ model for the ${k}_{s}$ prediction, which is straightforward and complementary to the previous ‘black box’ machine learning approaches. And the mathematical representation of the polynomial model is given by:

14 $${k}_{s}={{a}_{0}k}_{rms}{({a}_{1}+{a}_{2}ES)}^{{a}_{3}}({a}_{4}+{a}_{5}Sk\cdot Ku)$$

where ${a}_{i} \left(i=0, 1,\dots , 5\right)$ are the coefficients to be determined for the polynomial function.

Here the curve-fit function from the optimize module in the Scipy library is adopted to find the optimized values of coefficients. It uses the Levenberg–Marquardt algorithm which is an iterative algorithm used for solving nonlinear least square problems. This algorithm combines two optimization methods, namely, gradient descent and the Gauss–Newton method, aiming to provide robust and efficient converge performance under various conditions. The optimized coefficients are shown in Table 4:

**Table 4:** Optimized values of ${a}_{i}$ for the polynomial function.

${a}_{0}=$ 0.8878	${a}_{1}=$−0.1987	${a}_{2}=$ 2.5543
${a}_{3}=$ 0.3437	${a}_{4}=$ 5.0568	${a}_{5}=$ 0.3626

and the prediction quality of the optimized polynomial function can be achieved through the evaluation metrics, resulting in a calculated MAE is 0.0608, MSE is 0.0074, and MAPE is 38.87%.

Then we can summarize and compare the performance of the preliminary roughness correlation formulas with the data-driven methods, including the optimized ‘white box’ model and PSO-BPNN, and the comparison is conducted based on the evaluation metrics of MAE, MSE and MAPE:

It can be seen from the evaluation metrics that the accuracy of this optimized explicit prediction model is relatively lower than that of the PSO-BP method, but is more promising than results from other selected roughness correlation formulas under the given dataset. Another thing to be noted is that MAPE values for roughness correlation formulas, optimized polynomial model and neural networks are relatively large for this particular problem, that’s because ${k}_{s}$ values in the dataset are close to zero which will be heavily penalized when calculating with the MAPE equation. Overall, it is apparent from Table 5 that the PSO-BPNN outperforms both the selected roughness correlation formulas and the optimized polynomial model among the evaluation metrics of MAE, MSE and MAPE. And it can be concluded that the PSO-BPNN model is a rather effective and accurate method to predict the equivalent sand-grain roughness ${k}_{s}$ in the fully rough regime at various roughness types.

**Table 5:** ${k}_{s}$ prediction statistics comparison.

	Musker et al.	Flack et al.	Kuwata et al.	‘White box’ model	PSO-BPNN
MAE	0.1316	0.1034	0.0729	0.0608	0.0390
MSE	0.0255	0.0183	0.0141	0.0074	0.0026
MAPE	76.22%	51.66%	56.91%	38.87%	28.12%

### Uncertainty analysis

Recognizing and quantifying uncertainty is crucial for making informed decisions and understanding the reliability of PSO-BPNN’s prediction toward ${k}_{s}$. One of the most effective methods to visualize and comprehend the uncertainty of model predictions is through Confidence Intervals (CI). Thus, by using previous results from the PSO-BP model and computing CI with the predicted ${k}_{s}$ values, we obtain a probabilistic range wherein the true ${k}_{s}$ value is likely to fall, with a specified level of confidence. This approach not only provides a quantitative measure of the model's uncertainty but also offers a visual tool to grasp the potential variations in predictions.

**Figure 7:**
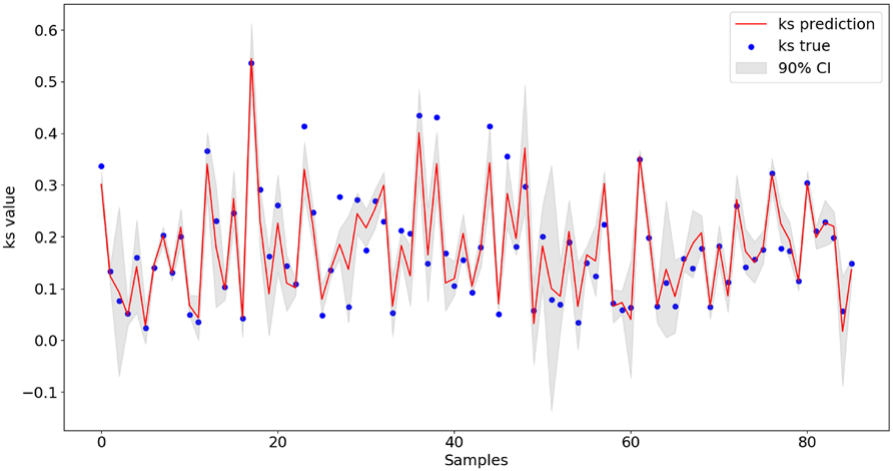
90% Confidence Interval for PSO-BPNN.

For our study, a 90% confidence level was chosen, which indicates that we are 90% confident that the true ${k}_{s}$ value lies within the computed intervals. The confidence interval is calculated using the following formula:

15 $$CI=\overline{x}\pm t\times \frac{\sigma }{\sqrt{n} }$$

where $\overline{x }$ is the sample mean, $t$ value is a critical factor determined by confidence level and degrees of freedom (approximately 2.13 in this case), $\sigma$ denotes the sample standard deviation and $n$ is the sample size. The results of this calculation are depicted in Fig. 7.

From the confidence interval plot, it’s evident that most of the ${k}_{s}$ true values fall within the 90% confidence interval, suggesting that the PSO-BPNN model is generally accurate and reliable for most predictions. And the model’s ability to follow the general trend of the ${k}_{s}$ true values indicate a good understanding of the underlying data patterns and relationships between surface parameters and ${k}_{s}$. However, regions with noticeably broader confidence intervals highlight where the model exhibits higher uncertainty. This increased uncertainty may be attributed to outliers or regions in the input space where the model is less trained. Yet, larger ${k}_{s}$ values tend to deviate from the confidence interval, aligning with the observation before that the PSO-BP model has less prediction accuracy for large ${k}_{s}$.

While the PSO-BP model demonstrates commendable robustness, areas of heightened uncertainty and deviations in larger ${k}_{s}$ values underscore potential avenues for improvement. It's important for future work to probe deeper into these regions of increased uncertainty and train with more large ${k}_{s}$ values, enhancing the model's comprehensiveness and predictive accuracy across the entire problem domain.

## Discussion

The PSO-BPNN model results reveal its efficiency in predicting the equivalent sand-grain roughness ${k}_{s}$ and demonstrate that neural networks can be successfully utilized for this kind of prediction task. In addition, the implementation of PSO in conjunction with BP significantly improves the prediction results, as evidenced by the reduction in MAE, MSE and MAPE by 36.38%, 59.38% and 38.96% respectively, when compared to the BP model alone. The PSO algorithm helps to optimize the weights and biases in the BP neural network, and this process can be considered as an implicit form of feature selection which enhances the model’s ability to accurately represent the dataset. This finding substantiates the effectiveness of PSO in optimizing of BP, underscoring the PSO-BP model as a more efficient method for the prediction of ${k}_{s}$.

In fact, the development of a predictive model for ${k}_{s}$ with arbitrary roughness types, based on multiple surface topography from a selected dataset, is labeled a complex nonlinear regression problem that is well-suited to the ML technique. Since various surface roughness yield different flow phenomena and makes a general physical model difficult to achieve, the ML technique takes no account of the physical modeling concept or observed phenomena within roughness sublayers for each case, nor is it affected by the lack of orthogonality among surface parameters chosen as the independent variables toward ${k}_{s}$. The flexibility of ML enables it to mimic models with many degrees of freedom or multivariate problems, and its feature selection property allows for the calibration of polynomial coefficients across different regions of the surface parameter space, while in a more thoroughly manner. In contrast, the reduced accuracy in roughness correlation formulas and optimized polynomial model can be attributed to the limitations inherent to low-order polynomial functions, which may not adequately capture the dependence of ${k}_{s}$ on surface parameters. And this is due to the constraint that each coefficient in the polynomial function must maintain a uniform value across the entire surface-parameter space. Even though, the optimized ‘white box’ polynomial model, which balances the trade-off between predictive accuracy and transparency, could serve as a valuable baseline for future studies aiming to parameterize ${k}_{s}$ explicitly across a broad spectrum of roughness types.

## Conclusion

In this study, we first utilized datasets from the fully rough region to devise the predictions for ${k}_{s}$ according to the analyzed most important surface parameters, namely the root-mean-square roughness height ${k}_{rms}$, effective slope $ES$, skewness $Sk$ and kurtosis $Ku$ with both BP and PSO-BP methods. Next, a Random Forest analysis is performed to find the appropriate roughness correlation formulas corresponding to the feature importance result. It also reveals that ${k}_{rms}$ emerges as the most influential feature toward the parameterization of ${k}_{s}$, followed sequentially by $Sk$, $ES$ and $Ku$ in descending order of importance. In addition, a ‘white box’ polynomial function for ${k}_{s}$ prediction is proposed based on the curve-fit technique. We also conducted an uncertainty analysis to assess the reliability of the PSO-BP model’s predictions. It turns out the PSO-BP method offers a better accuracy among the existing roughness correlation formulas, the traditional BP method as well as the explicit optimized polynomial functions. Furthermore, the training time for the PSO-BP method is less than 2 min with few hyperparameters to tune, making it a time-saving and efficient ML approach.

We therefore conclude that the PSO-BP method yields high-fidelity predictions of ${k}_{s}$ for turbulent flows over a wide range of rough surfaces and outperforms other listed methods, hence providing efficient and reliable inputs for further roughness-related CFD simulations or other engineering applications. In order to improve the model accuracy in further study, more data sets containing surface parameters and corresponding equivalent sand-grain roughness, especially larger ${k}_{s}$ values are needed to train the model, and enhancement could also be achieved by incorporating additional roughness parameters as input features, such as solidity, porosity and so on.

### Supplementary Information

Supplementary Information.

## Data Availability

The dataset examined in the current study is presented in this paper and supplementary materials.
