# Supplementary material for: Data-driven prediction of the equivalent sand-grain roughness
Source: Sci Rep. 2023 Nov 4;13:19108. doi: 10.1038/s41598-023-46564-4 (PMC10625589; doi:10.1038/s41598-023-46564-4)
Supplement: Supplementary file 1 — Supplementary Information. [file 41598_2023_46564_MOESM1_ESM.docx]

**Supplementary Materials**

A. Dataset and model

The full dataset used to train the PSO-BP and BP models, consisting of surface parameters and corresponding $k_{s}$

| $\boldsymbol{k}_{\boldsymbol{rms}}$ | $\boldsymbol{ES}$ | $\boldsymbol{Sk}$ | $\boldsymbol{Ku}$ | $\boldsymbol{k}_{\boldsymbol{s}}$ |
| --- | --- | --- | --- | --- |
| 0.0219 | 0.5949 | -0.1671 | 1.6007 | 0.0644 |
| 0.0326 | 0.9158 | 0.1093 | 1.4355 | 0.1244 |
| 0.0218 | 0.5876 | -0.0874 | 1.5898 | 0.0589 |
| 0.0223 | 0.5114 | 0.5600 | 2.2435 | 0.1362 |
| 0.0298 | 0.8612 | 0.8702 | 2.6269 | 0.3223 |
| 0.0214 | 0.5168 | 0.5141 | 2.2923 | 0.1311 |
| 0.0298 | 0.4979 | 0.9671 | 2.8736 | 0.2689 |
| 0.0437 | 0.7579 | 1.1501 | 3.1762 | 0.5364 |
| 0.0315 | 0.5076 | 0.7575 | 2.6415 | 0.2717 |
| 0.0217 | 0.6130 | 0.7078 | 1.8403 | 0.1406 |
| 0.0182 | 0.3337 | 1.6456 | 4.0941 | 0.1571 |
| 0.0304 | 1.1370 | -0.5377 | 1.8731 | 0.0767 |
| 0.0310 | 0.5372 | 0.9822 | 2.3078 | 0.2598 |
| 0.0259 | 0.3068 | 1.8486 | 4.8394 | 0.2465 |
| 0.0207 | 0.4870 | 0.7322 | 3.4216 | 0.1582 |
| 0.0169 | 0.3108 | 1.3431 | 4.1256 | 0.1057 |
| 0.0161 | 0.2189 | 2.1683 | 7.7277 | 0.1027 |
| 0.0287 | 0.4556 | 0.5910 | 2.8296 | 0.2768 |
| 0.0249 | 0.3046 | 1.4673 | 4.3466 | 0.1751 |
| 0.0265 | 0.2542 | 2.3707 | 8.7396 | 0.2603 |
| 0.0139 | 0.2124 | -0.0712 | 1.5051 | 0.0653 |
| 0.0210 | 0.1581 | -0.0308 | 1.4991 | 0.0712 |
| 0.0210 | 0.3162 | -0.0709 | 1.5031 | 0.1120 |
| 0.0203 | 0.9168 | -0.2033 | 1.5426 | 0.0640 |
| 0.0114 | 0.3704 | 0.3777 | 2.7840 | 0.0487 |
| 0.0184 | 0.4280 | 0.3231 | 2.6859 | 0.1088 |
| 0.0162 | 0.2803 | 0.0372 | 2.9768 | 0.0504 |
| 0.0171 | 0.4252 | 0.4758 | 2.9703 | 0.0930 |
| 0.0184 | 0.0967 | 0.2001 | 3.4927 | 0.0241 |
| 0.0228 | 0.2493 | 2.1006 | 5.5686 | 0.1499 |
| 0.0204 | 0.7202 | -0.7109 | 3.8425 | 0.0520 |
| 0.0205 | 0.7401 | -0.3380 | 3.1589 | 0.0500 |
| 0.0185 | 0.6177 | -1.1689 | 5.2921 | 0.0580 |
| 0.0165 | 0.5870 | -1.4448 | 5.4214 | 0.0560 |
| 0.0175 | 0.6362 | -0.7384 | 3.7139 | 0.0430 |
| 0.0126 | 0.6209 | -0.6874 | 3.8541 | 0.0350 |
| 0.0264 | 0.1810 | 0.1074 | 2.9405 | 0.0530 |
| 0.0318 | 0.1624 | 0.0934 | 2.9670 | 0.0340 |
| 0.0455 | 0.2268 | -0.0797 | 2.9891 | 0.0650 |
| 0.0877 | 0.4466 | -0.0651 | 2.9253 | 0.2000 |
| 0.0881 | 0.4336 | -0.6595 | 3.2739 | 0.1600 |
| 0.0885 | 0.4551 | -0.3512 | 3.0406 | 0.1800 |
| 0.0895 | 0.4448 | 0.3458 | 3.0505 | 0.2450 |
| 0.0866 | 0.3909 | 0.8117 | 3.5588 | 0.4350 |
| 0.1142 | 0.5569 | -0.0662 | 2.7937 | 0.2300 |
| 0.0450 | 0.8800 | 0.2100 | 2.5700 | 0.3659 |
| 0.0450 | 0.8800 | 0.2100 | 2.5800 | 0.4311 |
| 0.0450 | 0.6000 | 0.2100 | 2.6200 | 0.4140 |
| 0.0450 | 0.4000 | 0.2000 | 2.6200 | 0.3551 |
| 0.0450 | 0.3000 | 0.2000 | 2.6200 | 0.2912 |
| 0.0450 | 0.2000 | 0.2000 | 2.6200 | 0.1481 |
| 0.0450 | 0.4000 | -0.3400 | 2.6200 | 0.1395 |
| 0.0450 | 0.8800 | 0.2100 | 1.9200 | 0.2232 |
| 0.0450 | 0.8900 | 0.2200 | 1.8800 | 0.3038 |
| 0.0450 | 0.8900 | 0.2100 | 1.8800 | 0.3362 |
| 0.0450 | 0.8900 | 0.2000 | 1.8900 | 0.2286 |
| 0.0342 | 0.4700 | 0.0100 | 3.2800 | 0.1417 |
| 0.0555 | 0.4800 | -0.0100 | 3.2500 | 0.2967 |
| 0.0742 | 0.4900 | 0.0000 | 3.2700 | 0.4133 |
| 0.0347 | 0.5200 | -1.0000 | 26.8000 | 0.0783 |
| 0.0342 | 0.5200 | -0.3200 | 6.1500 | 0.1117 |
| 0.0338 | 0.4400 | 0.3000 | 2.2200 | 0.1733 |
| 0.0335 | 0.3100 | 1.0000 | 2.4400 | 0.2283 |
| 0.0184 | 0.0790 | 3.9560 | 18.0000 | 0.0696 |
| 0.0218 | 0.1410 | 2.7560 | 9.4600 | 0.1149 |
| 0.0269 | 0.3170 | 1.4090 | 3.5200 | 0.2028 |
| 0.0287 | 0.3910 | 1.0950 | 2.7000 | 0.1975 |
| 0.0300 | 0.4570 | 0.8690 | 2.2600 | 0.1773 |
| 0.0304 | 0.5090 | 0.7090 | 2.0100 | 0.1741 |
| 0.0311 | 0.5640 | 0.5610 | 1.8400 | 0.1553 |
| 0.0312 | 0.6340 | 0.3930 | 1.7100 | 0.1822 |
| 0.0184 | 0.0790 | 3.9210 | 17.7000 | 0.0662 |
| 0.0228 | 0.1400 | 2.7090 | 9.1500 | 0.1335 |
| 0.0278 | 0.3100 | 1.3110 | 3.2000 | 0.2103 |
| 0.0302 | 0.3790 | 0.9600 | 2.3600 | 0.1980 |
| 0.0310 | 0.4400 | 0.6900 | 1.9100 | 0.1776 |
| 0.0317 | 0.4860 | 0.4980 | 1.6900 | 0.1811 |
| 0.0332 | 0.5320 | 0.2940 | 1.5400 | 0.2120 |
| 0.0315 | 0.5910 | 0.0350 | 1.5100 | 0.1486 |
| 0.0600 | 0.2060 | -0.1900 | 2.3700 | 0.1900 |
| 0.0700 | 0.2410 | 0.0100 | 5.1000 | 0.3494 |
| 0.0600 | 0.2030 | -0.2960 | 2.2000 | 0.2000 |
| 0.0330 | 0.2200 | 0.0100 | 2.9000 | 0.1622 |
| 0.0280 | 0.3400 | 0.0100 | 3.0000 | 0.1439 |
| 0.0280 | 0.3400 | 0.0100 | 2.9800 | 0.1688 |
| 0.0310 | 0.3500 | -0.0200 | 2.9400 | 0.2062 |

And the trained PSO-BP model can be accessed online in the author’s GitHub repository at <https://github.com/mhr930608/PSO-BP-for-Surface-Roughness.git>.

B. Mathematical expressions for surface parameters

| Root-mean-square height | $k_{rms}=\left( \frac{1}{S}\int_{S} k^{2}dS \right)^{\frac{1}{2}}$ |  |
| --- | --- | --- |
| Effective slope | $ES=\frac{1}{S}\int_{S} \left\vert\frac{\partial k}{\partial x} \right\vert dS$ |  |
| Frontal solidity | $\lambda_{f}=\frac{A_{f}}{A_{t}}$ |  |
| Roughness density parameter | $\Lambda_{s}={\lambda_{f}}^{-1}\frac{A_{f}}{A_{w}}$ |  |
| Plan solidity | $\lambda_{p}=\frac{A_{p}}{A_{t}}$ |  |
| Skewness | $Sk=\frac{1}{{k_{rms}}^{3}}\int_{S} k^{3}dS$ |  |
| Kurtosis | $Ku=\frac{1}{{k_{rms}}^{4}}\int_{S} k^{4}dS$ |  |

where $k$ represents the surface height, $S$ denotes the wall-projected surface area, and $x$ refers to the streamwise direction. Additionally, $A_{f}$ is the projected frontal area of the roughness elements, $A_{t}$ describes the total plan area of the domain, $A_{w}$ is the total windward wetted surface area, and $A_{p}$ is the roughness elements plan area. Furthermore, solid volume fraction $\phi$ can be interpreted as a volume-based generalization of plan solidity.
